# TROP2 promotes proliferation, migration and metastasis of gallbladder cancer cells by regulating PI3K/AKT pathway and inducing EMT

**DOI:** 10.18632/oncotarget.16789

**Published:** 2017-04-03

**Authors:** Xinxing Li, Shifeng Teng, Yanyan Zhang, Weigang Zhang, Xianwen Zhang, Kai Xu, Houshan Yao, Jun Yao, Haolu Wang, Xiaowen Liang, Zhiqian Hu

**Affiliations:** ^1^ Department of General Surgery, Changzheng Hospital, The Second Military Medical University, Shanghai 200003, China; ^2^ Therapeutics Research Centre, School of Medicine, The University of Queensland, Princess Alexandra Hospital, Woolloongabba QLD 4102, Australia

**Keywords:** gallbladder cancer, TROP2, AKT, EMT

## Abstract

The human trophoblast cell surface antigen 2 (TROP2) is overexpressed in many cancers. However, its effect on proliferation, migration and metastasis of gallbladder cancer remains unclear. In this study, we found that TROP2 was highly expressed in gallbladder cancer. Overexpression of TROP2 was associated with poor prognosis. Knockdown of TROP2 in gallbladder cancer cell lines strongly inhibited the cell proliferation, clone formation, invasion and migration *in vitro*, while TROP2 overexpression had opposite effects. In addition, knockdown of TROP2 increased the expression of total PTEN, p-PTEN and PDK-1 but reduced p-AKT via PI3K/AKT pathway. TROP2 downregulation also inhibited vimentin and increased E-cadherin expression during epithelial-mesenchymal transition (EMT). Moreover, gallbladder cancer cells with TROP2 knockdown formed smaller xenografted tumors *in vivo*. In consistent with *in vitro* results, TROP2 inhibition decreased Akt phosphorylation, increased PTEN expression and postponed EMT of gallbladder cancer cells *in vivo*. In conclusion, we revealed that TROP2 promoted the proliferation, migration and metastasis of gallbladder cancer cells by regulating PI3K/AKT pathway and inducing EMT. TROP2 could serve as a potential prognostic biomarker and therapeutic target for the clinical management of gallbladder cancer.

## INTRODUCTION

Gallbladder cancer (GBC) is the most common malignancy of biliary system with low curative resection rate (10–30%), low response rate to chemotherapy, and poor prognosis (5-year survival less than 5%) [1, 2, 3]. Thus, there is urgent need to reveal detailed signal pathways involved in GBC proliferation, migration and metastasis, which may provide potential prognostic biomarkers and therapeutic targets for the clinical management of GBC.

The human trophoblast cell surface antigen 2 (TROP2), a 36-kDa cell-surface glycoprotein, also known as EGP-1, M1S1, GA733-1, belongs to the TACSTD gene family [4–6]. TROP2 was first discovered in trophoblast cell which was invasive and metastasizing cell of the placenta from the outer layer of the blastocyst [7]. TROP2 was highly expressed in gastric cancer [8], cervical cancer [9], pancreatic cancer [10], colorectal cancer [11], lung cancer [12] and several types of stem cells [13]. TROP2 overexpression has been reported to be associated with poor survival, tumor aggressiveness and metastasis [6]. It was also involved in many signaling pathways of cell proliferation, survival and self-renewal [14]. Cubas *et al*. reported that TROP2 contributed to tumor pathogenesis via ERK/MAPK pathway [15]. Liu *et al*. found that TROP2 promoted the proliferation and invasion of cervical cancer cells by regulating ERK signaling pathway [9]. Although Chen *et al*. revealed that TROP2 overexpression was associated with poor prognosis of GBC [16], TROP2 signal pathways and possible mechanisms are still unclear in GBC proliferation, migration and metastasis. We have previously characterized the cancer stem-like cells from GBC cells [17], and investigated their multidrug resistance [18, 19]. In this study, we revealed that TROP2 promoted the proliferation, migration and metastasis of GBC cells by regulating PI3K/AKT pathway and inducing epithelial-mesenchymal transition (EMT). We suggest that TROP2 could serve as a potential prognostic biomarker and therapeutic target for the clinical management of GBC.

## RESULTS

### Overexpression of TROP2 was associated with poor prognosis of GBC

**Figure 1:**
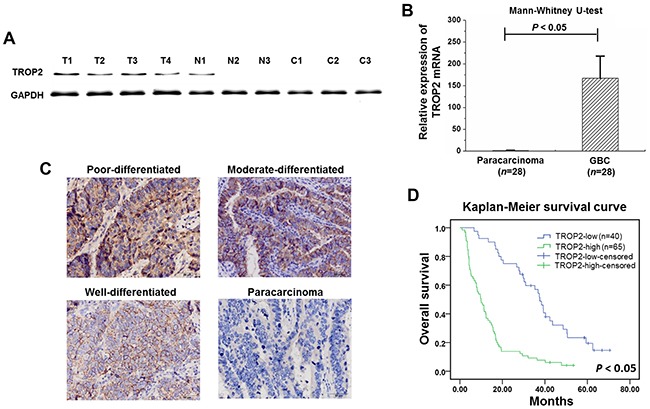
TROP2 expression in GBC samples **(A)** TROP2 protein was highly expressed in GBC, lower in paracarcinoma, and undetectable in chronic cholecystitis tissues (T: GBC, N: paracarcinoma, and C: chronic cholecystitis tissues). **(B)** TROP2 mRNA expression in GBC was significantly higher compared to paracarcinoma (167.06±50.71 VS 1.20±0.79, P<0.05). Columns, n=28; bars, S.D. P< 0.05, GBC compared with paracarcinoma. **(C)** TROP2 protein expression was higher in advanced GBCs revealed by IHC staining. No TROP2 expression was detected in paracarcinoma. **(D)** Kaplan-Meier survival curve showed that GBC patients with high TROP2 expression had a worse prognosis.

As shown in Figure 1A and 1B, TROP2 was highly expressed in GBC, lower expressed in paracarcinoma, and undetectable in chronic cholecystitis tissues of both protein and mRNA levels. TROP2 protein expression was higher in advanced GBCs revealed by histology examination (Figure 1C). Overexpression of TROP2 was closely correlated to gallstone presence, histological grade, tumor invasion, lymph node metastasis, TNM stage and poor survival (Table 1). Log-rank analysis showed that GBC survival was correlated to tumor size, histological grade, tumor invasion, lymph node metastasis and TROP2 expression (Table 2 and Figure 1D). Cox regression analysis confirmed that TROP2 expression and tumor invasion were two independent prognostic factors for GBC (Table 2). These results suggest that overexpression of TROP2 was associated with poor prognosis of GBC.

**Table 1:** Effects of TROP2 expression on clinicopathologic features of GBC

Parameter	Case	TROP2 expression		χ^2^	P value
		Low	High		
Sex					
Male	30	11	19	0.036	0.849
Female	75	29	46		
Age (years)					
≤60	37	11	26	1.695	0.193
>60	68	29	39		
With gallstone					
Present	79	36	43	7.558	**0.006**
Absent	26	4	22		
Tumor size					
≤3cm	38	11	27	2.113	0.146
>3cm	67	29	38		
Histological grade					
Well or morderate	54	33	21	24.973	**0.000**
Poor	51	7	44		
Tumor invasion (AJCC)					
Tis-T2	54	30	24	14.372	**0.000**
T3-T4	51	10	41		
Lymph node					
N0	58	33	25	19.422	**0.000**
N1-2	47	7	40		
TNM stage (AJCC)					
I-II	38	29	9	36.889	**0.000**
III-IV	67	11	56		

**Table 2:** Univariate and multivariate analysis of the prognostic factors for overall survival of GBC

Prognostic factors	Univariate analysis			Multivariate analysis		
	RR	95%CI	P value	RR	95%CI	P value
Sex	1.062	0.678-1.644	0.793			
Age (years)	1.118	0.772-1.809	0.443			
Associated gallstone	0.848	0.525-1.372	0.503			
Tumor size	0.006	0.001-0.045	**0.000**	0.000	0.169-4.183	0.846
Histological grade	0.084	0.045-0.157	**0.000**	0.387	0.124-1.206	0.102
Tumor invasion (AJCC)	0.103	0.056-0.189	**0.000**	0.352	0.183-0.678	**0.002**
Lymph node	0.113	0.063-0.200	**0.000**	0.795	0.271-2.281	0.670
TROP2 expression	0.259	0.163-0.412	**0.000**	0.463	0.274-0.782	**0.004**

### Expression of TROP2 in human GBC cell lines after RNA interference or plasmid transfection

**Figure 2:**
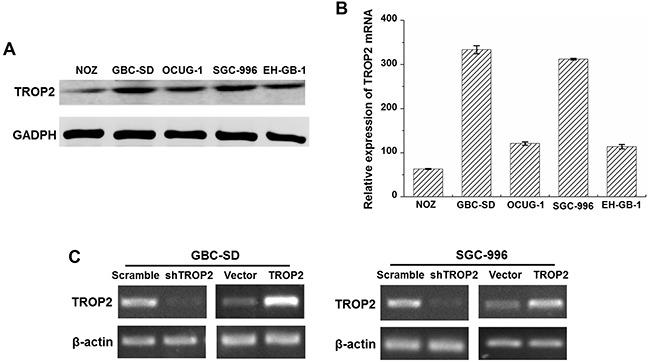
TROP2 expression in GBC cell lines **(A)** The TROP2 protein expression of human GBC cell lines NOZ, GBC-SD, OCUG-1, SGC-996 and EH-GB-1B. **(B)** The TROP2 mRNA expression of human GBC cell lines NOZ, GBC-SD, OCUG-1, SGC-996 and EH-GB-1B. **(C)** TROP2 mRNA expressions of GBC-SD and SGC-996 cells after RNA interference and plasmid transfection. Cells were transfected with scramble sh-RNA and empty vector as negative controls. Each experiment was repeated three times.

To further explore the function of TROP2 in GBC, its expression was examined in human GBC cell lines included NOZ, GBC-SD, OCUG-1, SGC-996 and EH-GB-1 using western blot and quantitative real-time PCR. As shown in Figure 2A and 2B, TROP2 were detected in all of GBC cell lines at both protein and mRNA levels. Since GBC-SD and SGC-996 cells had higher TROP2 expression among GBC cell lines, we selected these two cell lines for further experiments. We then used lentiviral-mediated shRNA plasmid transfection targeting TROP2 for stable transfection. The RT-PCR results showed that endogenous TROP2 mRNA expression was significantly inhibited at 48 h after transfection in both GBC-SD and SGC-996 cells (Figure 2C). The pcDNA 3.1-TROP(+) lentiviral vector was transfected into GBC-SD and SGC-996 cells for the gain-of-function study. RT-PCR results revealed that TROP2 expression was increased in stable transfection group compared to empty vector group (Figure 2C). Thus, we successfully established the GBC cells with TROP2 knockdown and overexpression for further experiments.

### Effects of TROP2 on proliferation and clone formation of GBC cells

**Figure 3:**
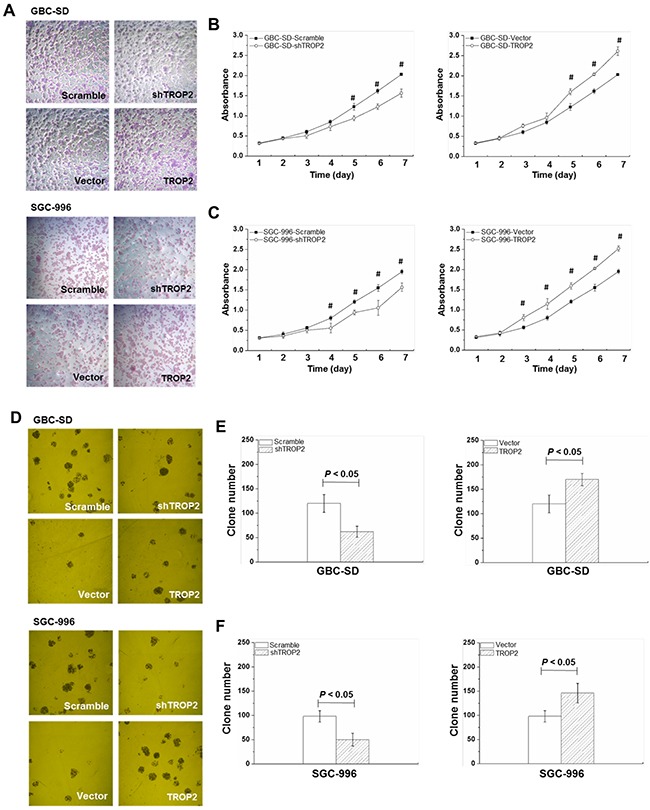
Effects of TROP2 on proliferation and clone formation of GBC-SD and SGC-996 cells **(A)** Microscope images of GBC-SD and SGC-996 cell growth after TROP2 knockdown and overexpression. **(B and C)** Growth curves of GBC-SD and SGC-996 cells after RNA interference or plasmid transfection. Graphs, mean of three experiments; bars, S.D. *P < 0.05, shTROP2 group compared with the control group. ^#^*P* < 0.05, TROP2 overexpression group compared with the control group. Each experiment was repeated three times. **(D)** Microscope images of GBC-SD and SGC-996 cell clone formation after TROP2 knockdown and overexpression. **(E and F)** The number of clone formation of GBC-SD and SGC-996 cells after RNA interference or plasmid transfection. Columns, mean of three experiments; bars, S.D. Each experiment was repeated three times.

To investigate the effects of TROP2 on proliferation and clone formation, we performed MTT and clone formation assays in GBC cells (Figure 3A and 3D). The GBC cells were cultured for 7 days and cell viabilities were tested each day. To exclude the possibility of off-target effect, the other two shRNAs (shTROP2-2 and shTROP2-3) were used for TROP2 knockdown (Supplementary Figure 1A, 1B and Supplementary Table 1). The scramble sh-RNA and an empty vector (Mock) were used as negative controls (Supplementary Figure 2). We found that the cell vitality significantly decreased in the shRNA-TROP2 group of GBC-SD cells from Day 5 (*P* < 0.05, Figure 3B and Supplementary Figure 2) and in the shTROP2-2 and shTROP2-3 groups from Day 3 (*P* < 0.05, Supplementary Figure 1C and 1D). We found that the number of colony formation in shRNA-TROP2 group of GBC-SD cells was lower than that of scramble group (*P* < 0.05, Figure 3E). Inversely, GBC-SD, NOZ and EH-GB-1 cells with TROP2 overexpression had stronger ability of proliferation and clone formation compared to the control groups (*P* < 0.05, Figure 3B, 3C, 3F and Supplementary Figure 3). TROP2 had the same effects on SGC-996 cell (Figure 3C, 3F, Supplementary Figure 1B and 1D).

### Effects of TROP2 on migration and invasion of GBC cells

**Figure 4:**
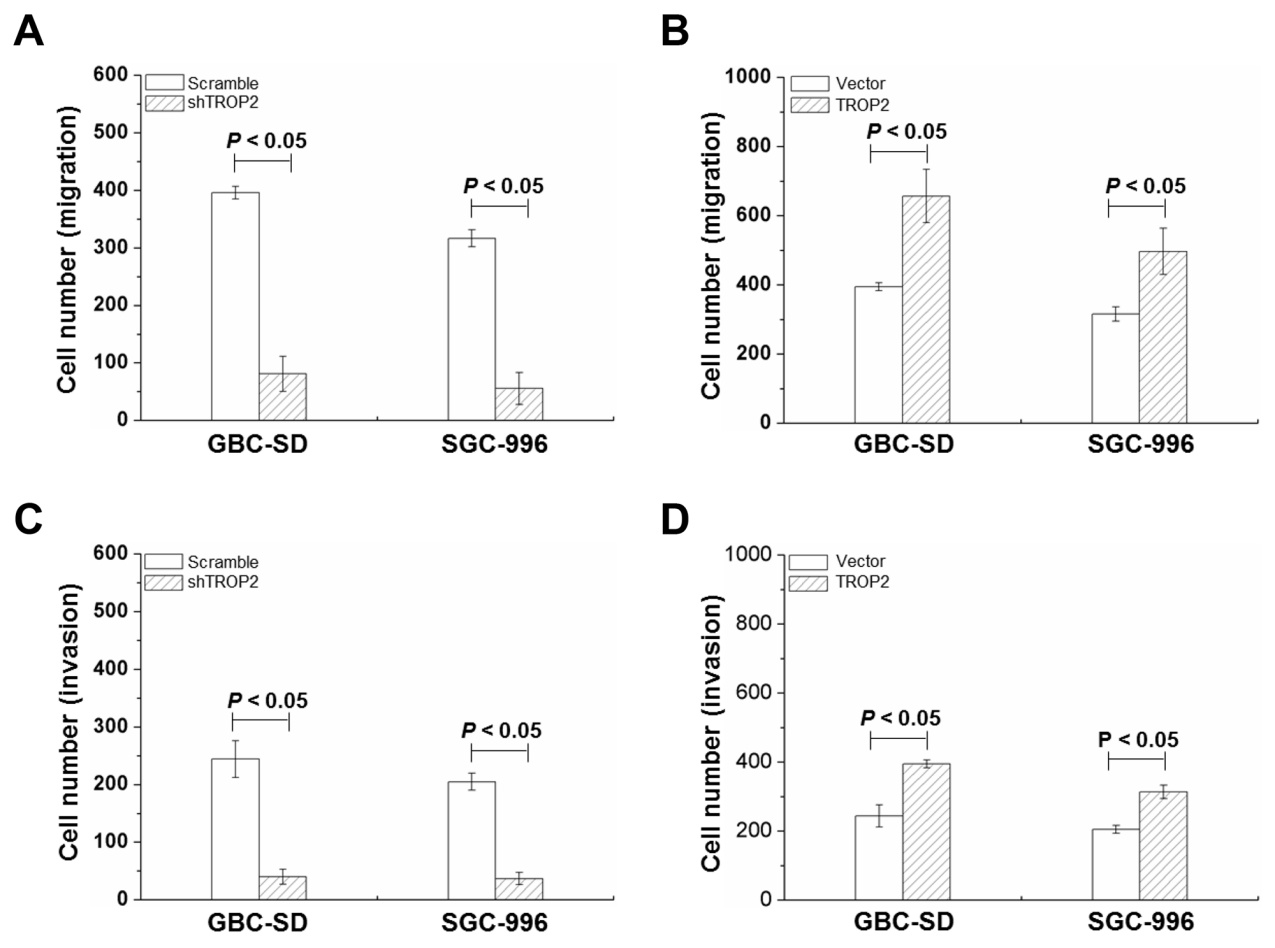
Effects of TROP2 on migration and invasion of GBC-SD and SGC-996 cells **(A and B)** Cell migration of GBC-SD and SGC-996 cells after RNA interference or plasmid transfection. **(C and D)** Cell invasion of GBC-SD and SGC-996 cells after RNA interference or plasmid transfection. Columns, mean of three experiments; bars, S.D. *P* < 0.05, TROP2 overexpression group compared with the empty vector group. Each experiment was repeated three times.

We further examined the effects of TROP2 on migration and invasion of GBC-SD and SGC-996 cells. As shown in Figure 4A, cell migration in shRNA-TROP2 groups was significantly lower than that of scramble groups (*P* < 0.05, Figure 4A), while TROP2 overexpression had the opposite effects (*P* < 0.05, Figure 4B). GBC cells with TROP2 downregulation had less invasive ability (*P* < 0.05, Figure 4C). As shown in Figure 4D and S3D, GBC cells showed higher invasive ability after increasing TROP2 expression. These results suggest that high expression of TROP2 can enhance the invasive and migration of GBC cells, which is a key fact in regulating the migration and invasion of GBC.

### Effects of TROP2 on xenografted tumor growth

**Figure 5:**
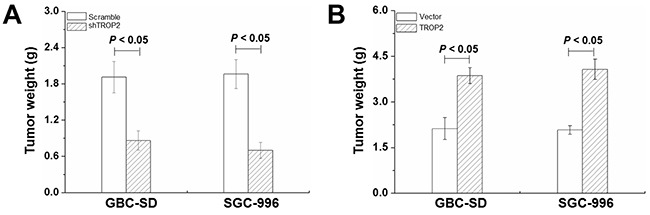
Effects of TROP2 on xenografted tumor growth **(A)** Tumor weight of GBC-SD and SGC-996 cells after RNA interference in nude mice at 6 weeks after implantation. **(B)** Tumor weight of GBC-SD and SGC-996 cells after plasmid transfection in nude mice at 6 weeks after implantation. Columns, n=8; bars, S.D. *P* < 0.05, shTROP2 group compared with the control group and TROP2 overexpression group compared with the control group.

To investigate the function of TROP2 *in vivo*, GBC cells were injected subcutaneously into 6-week-old BALB/c-nu/nu mice. As shown in Figure 5A, the tumor weight of shRNA-TROP2 groups significantly reduced compared to the scramble groups of both GBC-SD and SGC-996 cells (*P* < 0.05), while TROP2 overexpression had the opposite effects (*P* < 0.05, Figure 5B). These results indicate that TROP2 depletion can effectively suppress GBC growth *in vivo*.

### TROP2 regulates PI3K/AKT pathway and induces EMT *in vitro* and *in vivo*

**Figure 6:**
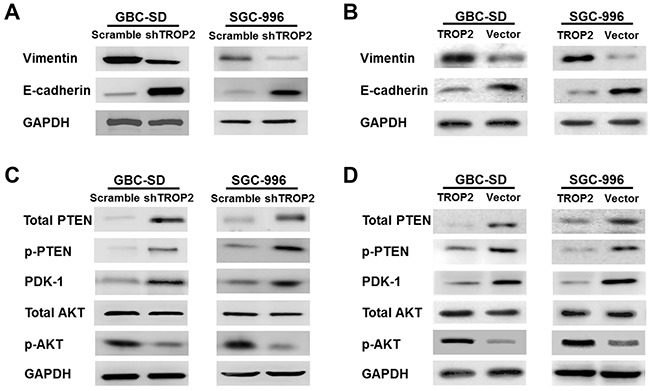
Effects of TROP2 on EMT and PI3K/AKT pathway in GBC-SD and SGC-996 cells *in vitro* **(A)** Knockdown of TROP2 expression inhibited vimentin and increased E-cadherin protein expression. **(B)** Overexpression of TROP2 increased vimentin and decreased E-cadherin protein expression. **(C)** Knockdown of TROP2 increased total PTEN, p-PTEN and PDK-1 protein expression, but inhibited p-AKT expression. **(D)** Overexpression of TROP2 decreased total PTEN, p-PTEN and PDK-1 protein expression, but increased p-AKT expression.

**Figure 7:**
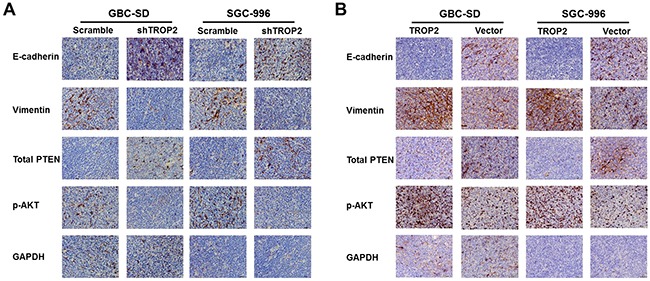
Effects of TROP2 on EMT and PI3K/AKT pathway *in vivo* **(A)** Immunohistochemical results of xenografted tumors showed that inhibition of TROP2 expression increased PTEN expression, decreased Akt phosphorylation, and postponed EMT progress (decreased vimentin and upregulated E-cadherin expression). **(B)** TROP2 overexpression decreased PTEN expression, and increased Akt phosphorylation and EMT progress (increased vimentin and downregulated E-cadherin expression).

Next, we explored the mechanisms of proliferation, migration and metastasis promoted by TROP2 in GBC. EMT is defined as the transformation of epithelial cells into spindle cells with the loss of membrane E-cadherin expression and the gain of mesenchymal markers such as vimentin, which promotes tumor initiation, progression and metastasis in human mammary epithelial cells. We found that downregulation of TROP2 decreased vimentin and increased E-cadherin expression both *in vitro* and *in vivo*, while TROP2 overexpression had the opposite effects (Figure 6A, 6B and 7).

It has been reported that Akt pathway was involved in cell proliferation and migration [20]. PTEN is a dual-phosphatase that negatively regulates AKT activity [21]. Loss of PTEN and activation of the PI3K/AKT pathway result in tumor progression and metastasis [20, 22–23]. We found that downregulation of TROP2 markedly decreased the expression of active Akt (phosphorylated at Thr308) *in vitro* (Figure 6C), while the expression of total Akt protein did not change significantly. TROP2 knockdown increased the expression of total PTEN, p-PTEN and PDK-1 (Figure 6C). Thus, downregulation of TROP2 can inhibit Akt phosphorylation and increase PTEN expression. In consistent with the *in vitro* results, TROP2 inhibition decreased Akt phosphorylation and increased PTEN expression *in vivo* (Figure 7A and 7B), while TROP2 overexpression had the opposite effects (Figure 6B and 6D). Altogether, our data suggest that TROP2 is involved in the PI3K/AKT pathway and induced EMT both *in vitro* and *in vivo*.

## DISCUSSION

Over the past 20 years, molecule-targeted drug has become an effective method in cancer therapy, especially for lung cancer and breast cancer [24–25]. However, until now, no specific targeted therapy has been developed for GBC. TROP2, originally identified in human trophoblast and choriocarcinoma cells, which was highly expressed in many cancers include pancreatic carcinoma [10], gastric carcinoma [8], lung carcinoma [12] and colorectal cancer [11]. Stoyanova *et al*. demonstrated that TROP2 controlled stem/progenitor self-renewal and tissue regeneration [26]. In this study, we found TROP2 was highly expressed in GBC, lower in paracarcinoma, and undetectable in chronic cholecystitis tissues at both protein and mRNA levels. The reason might be that TROP2 is an oncogene of GBC, which is remarkably associated with cancer development and progression [16]. Furthermore, overexpression of TROP2 was correlated to gallstone presence, histological grade, tumor invasion, lymph node metastasis, TNM stage and poor survival. Among them, gallstone has been considered as the important reason for the occurrence of GBC [27]. TROP2 expression was further confirmed as an independent prognostic factor for GBC by Cox regression. All these data suggest that TROP2 could serve as a potential prognostic biomarker and therapeutic target for the clinical management of GBC.

We also found that TROP2 is a key fact in proliferation, clone formation migration and invasion of GBC. The PI3K/AKT pathway governs many cellular processes, including cell growth, proliferation, metabolism and cellular architecture advantages [22–23]. By acting as a unique lipid phosphatase, PTEN acts as the major cellular suppressor of PI3K signaling and AKT activation [22]. PTEN negatively regulates PI3K/AKT signaling and is often inactivated by mutations (including deletions) in a variety of cancer types [22]. Over-activation of Akt has been recognized to induce oncogenic [20, 22–23] and modulate cell growth and survival in GBC [28, 29]. Roa *et al*. reported that loss of PTEN expression was associated with advanced GBC and poor prognosis [30]. Lunardi *et al*. identified the role of aberrant PI3K pathway activation in gallbladder tumorigenesis [31]. Furthermore, Goldstein *et al*. found that TROP2 expression was significantly increased during tumorigenesis caused by aberrant PI3K signaling [32]. Similar to these findings, we revealed that TROP2 regulates PI3K/AKT pathway and induces EMT both *in vitro* and *in vivo*. In contrast, Lin *et al*. observed low TROP2 expression in lung adenocarcinoma tissues compared to their normal counterparts [12]. They found TROP2 could attenuate IGF-1R signalling-mediated AKT activation through a direct binding of IGF1. This might be due to either the loss of heterozygosity or hypermethylation of the CpG island DNA of TROP2 upstream promoter region, which have not been fully found in the other cancers.

EMT is a morphological change of tissues/cells from an epithelial form to a fibroblast-like mesenchymal form [33–34], which has been regarded as one of the most important processes in cancer invasion and metastasis [33]. During this process, epithelial cells lose their properties such as E-cadherin and acquire mesenchymal phenotypes such as vimentin [35–36]. Chen *et al*. found that high TROP2 expression was significantly associated with loss of E-cadherin and acquisition of vimentin [16]. Our data showed that TROP2 downregulation could inhibit vimentin expression and upregulate E-cadherin expression both *in vitro* and *in vivo*, while TROP2 overexpression had the opposite effects. These results indicate that TROP2 plays an important role in regulating the process of EMT in GBC invasion and metastasis. Further study is needed to clarify whether Akt activation or EMT is affected by PTEN or E-cadherin.

In conclusion, we found that overexpression of TROP2 was associated with poor prognosis of GBC. TROP2 promotes the proliferation, migration and metastasis of GBC cells by regulating PI3K/AKT pathway and inducing epithelial-mesenchymal transition. We suggest that TROP2 could serve as a potential prognostic biomarker and therapeutic target for the clinical management of GBC.

## MATERIALS AND METHODS

### Patients and clinicopathological data

The specimens were obtained between 2003 and 2010 from 105 patients with pathologically confirmed GBC, who underwent primary tumor resection at Changzheng Hospital affiliated with the Second Military Medical University (Shanghai, China). Among the 105 GBC cases, there were 30 males and 75 females with ages ranging from 31 to 92 years (mean age: 64.82 years). All specimens and fresh tissue samples had been confirmed by pathological diagnosis and were staged according to the 7th AJCC-TNM classification of malignant tumors. The median follow-up period was 22.23 months (range, 1–70.5 months). In this study, no patients received chemotherapy or radiotherapy before surgery. The clinicopathological information and patients’ medical history were documented during post-operative follow up. Prior to our scientific research, patient's consent was obtained and this study was approved by the ethics committee of the Second Military Medical University.

### Western blot analysis

Cellular proteins were extracted using radio-immunoprecipitation assay (RIPA) buffer according to the method described by Cubas et al. [15]. Equal amount of proteins was electrophoresed on 12% SDS-polyacrylamide gel and transferred to a nitrocellulose membrane. The membrane was incubated for 1 h in blocking buffer (5% low-fat milk powder in TBS containing 0.1% Tween) and then incubated with the mouse antibody against human TROP2 (R&D Systems, Inc., Minneapolis, USA), vimentin, E-cadherin, PTEN, p-PTEN, PDK-1, AKT, and p-AKT (Abcam, Cambridge, UK) at 4°C for overnight and horseradish peroxidase-conjugated goat anti-mouse immunoglobulin (Sigma, St. Louis, MO, USA) for 1 h before detected by an enhanced chemiluminescence (ECL) system. GADPH was used as the loading control and anti-GADPH antibody was obtained from Cell Signaling Technology (Danvers, USA).

### Quantitative real-time PCR and RT-PCR

Total mRNA was extracted using Trizol reagent (Invitrogen, Carlsbad, CA, USA) according to the instruction of the manufacturer as previously used by Li et al. [37]. The cDNA was reverse-transcribed from 2 ug total RNA. Detection of PCR products was performed on a Light Cycler system (Roche Applied Science, Basel, Switzerland) using the SYBR Green I kit (TaKaRa Biotechnology, Dalian, China), according to the manufacturer's instructions. Each sample was done in triplicate. The internal control for real-time PCR was β-actin. The relative expression level of the target gene was calculated and normalized to the relative expression detected in the corresponding control cells, which was defined as 1.0.

Total mRNA was extracted using Trizol reagent (Invitrogen, Carlsbad, CA, USA) according to the instruction of the manufacturer. The cDNA of TROP2 expression was reverse transcribed from 2 ug total RNA. β-actin was used as an internal control. The PCR was performed as follows: denaturation for 5min at 95°C, 30 cycles of 95°C for 30s, 55°C for 45s and 72°C for 30s, then extended for 10 min at 72°C.

### Immunohistochemistry

Immunohistochemistry (IHC) staining was performed using the standard immunoperoxidase staining procedure, and TROP2 expression in the specimens was evaluated according to the methods described by Fong et al. [10]. TROP expression was evaluated for each tissue sample by calculating a total immunostaining score as the product of a proportion and intensity score. The proportion score described the estimated fraction of positive stained tumor cells (0, none; 1, <10%; 2, 10-50%; 3, 51-80%; 4, >80%). The intensity score represented the estimated staining intensity (0, no staining; 1, weak; 2, moderate; 3, strong). The total score ranged from 0 to 12. The high expression of TROP2 was defined as a total score of more than 4 described by Fong et al. [10]. Expressions of vimentin, E-cadherin, p-AKT and PTEN in animal transplanted tumor paraffin tissue sections was adopted following the above experiment method.

### Cell culture

The human GBC cell lines NOZ, GBC-SD, OCUG-1, SGC-996 and EH-GB-1 were purchased from the Shanghai Cell Institute National Cell Bank. NOZ and SGC-996 were cultured in RPMI-1640 medium (GibcoBRL, Gaitherburg, MD, USA). The remaining cell lines were cultured in Dulbecco's Modified Eagles Medium (DMEM) (GibcoBRL, Gaitherburg, MD, USA). The media were supplemented with antibiotics and 10% newborn calf serum. Cells were cultured in a humidified atmosphere with 5% CO2 at 37 C.

### RNA interference and plasmid transfection

As described by Zhang et al. [38], for lentivirus-mediated silencing of TROP2, the short hairpin RNA (shRNA) effectively targeted human TROP2 to acquire the stabilized expression. The recombinant plasmid pGensil1.1-TROP2-shRNA (including shTROP2, shTROP2-2 and shTROP2-3) was provided by Dr. Dai from Jiangsu University. The GBC-SD and SGC-996 cells were infected with concentrated vector according to the manufacturer's instructions. For the generation of stable GBC-SD and SGC-996 cells overexpressing human Trop2, a pcDNA 3.1-TROP(+) vector (a kind gift from Dr. Yang from Shandong University) was utilized. For transfection, cells were seeded into 6-well plates and expected to be 50% confluency next day. Cells transfected with scrambled shRNA or empty vectors were considered as negative control. Lipofectamine 2000 (Invitrogen) was used for transfection according to the manufacturer's instructions. The relative levels of TROP2 in the transfected cells were examined by RT-PCR. Functional assays were performed 48 h after transfection.

### Cell proliferation and clone formation assays

For cell proliferation study, growth curves of GBC-SD and SGC-996 cells were determined by the MTT assay (Sigma-Aldrich Corp. St. Louis, MO USA), as described by Li et al. [17]. Approximately 2.0×10^3^ cells per well were seeded in 96-well plates. After incubation time from day 1 to day 7, 10 μL MTT (5 mg/ml) was added to each well and incubated at 37 C for 4 h. Afterwards, the culture medium was removed and 100 μL dimethyl sulphoxid (DMSO) (Invitrogen, Carlsbad, CA, USA) was added to each well. After shaking thoroughly for 10 min, the absorbance of each well was determined with a spectrophotometer at a wave length of 570 nm. Triplicate wells were used for each group. To examine clonogenic ability, GBC-SD and SGC-996 cells were plated at 500 cells per well in six-well culture dishes in culture media with 10% newborn calf serum. Triplicate wells were performed for each group. After 2 weeks, clones with ≥50 cells were scored under the microscope.

### Cell migration and invasion assays

As described by Li et al. [17], a transwell assay was used to examine cell migration and invasion. 2×10^4^ cells were plated in 300 μL of serum-free medium. The upper chamber is made with the Boyden chamber containing an 8 μm pore size membrane (BD Biosciences, San Jose, CA). In cell invasion studies the membranes were pre-coated with Matrigel (1 mg/ml; BD Biosciences, San Jose, CA). In the lower chamber, 500 μL of culture media containing 0.2% FBS were added. After 24 h, the chamber was removed, fixed, and stained with trypan blue. Cells in the upper chamber were removed using the alcohol. Cells migrating through the membrane and cells invading the matrix were photographed in three randomly-selected fields and counted in average.

### Tumorigenicity assay *in vivo*

Approval for the animal experiments was obtained from the ethics committee of the Second Military Medical University. To explore the tumorigenic capacity, 2×10^6^ cells, sorted and suspended in 100 μL culture media containing 0.2% FBS, were injected subcutaneously into 6-week old BALB/c-nu/nu mice. The mice were monitored every week for palpable tumor formation. After 6 weeks, the mice were sacrificed. Tumors were then dissected and weighed. Tumor tissues were fixed by methanol for subsequent immunohistochemical experiment.

### Statistical analysis

Statistical analyses were performed using SPSS Statistics 18.0 software (SPSS Inc., Chicago, IL, USA) and results were considered statistically significant at p<0.05. Data were shown as mean values ± S.D., and some of the data were displayed in the form of chart. Differences between groups were evaluated by Student's t test or one-way ANOVA, and continuous variables were evaluated using Wilcoxon rank and inspection. Pearson chi-square test or Fisher's exact test was used for analysis between the degree of staining and clinical parameters. Kaplan–Meier curves were plotted to describe the survival information by the log-rank test. Multivariate analysis was performed using a Cox's proportional hazards regression model.

## SUPPLEMENTARY MATERIALS FIGURES AND TABLES


