# Correction: TROP2 promotes proliferation, migration and metastasis of gallbladder cancer cells by regulating PI3K/AKT pathway and inducing EMT

**DOI:** 10.18632/oncotarget.27299

**Published:** 2019-11-05

**Authors:** Xinxing Li, Shifeng Teng, Yanyan Zhang, Weigang Zhang, Xianwen Zhang, Kai Xu, Houshan Yao, Jun Yao, Haolu Wang, Xiaowen Liang, Zhiqian Hu

**Affiliations:** ^1^ Department of General Surgery, Changzheng Hospital, The Second Military Medical University, Shanghai 200003, China; ^2^ Therapeutics Research Centre, School of Medicine, The University of Queensland, Princess Alexandra Hospital, Woolloongabba QLD 4102, Australia

**This article has been corrected:** Due to errors in image processing, Figure 2C contains an image from the wrong cell line (GBC-SD cells). The corrected Figure 2C is shown below. The authors declare that these corrections do not change the results or conclusions of this paper.

Original article: Oncotarget. 2017; 8:47052–47063. 47052-47063. https://doi.org/10.18632/oncotarget.16789

**Figure 2:**
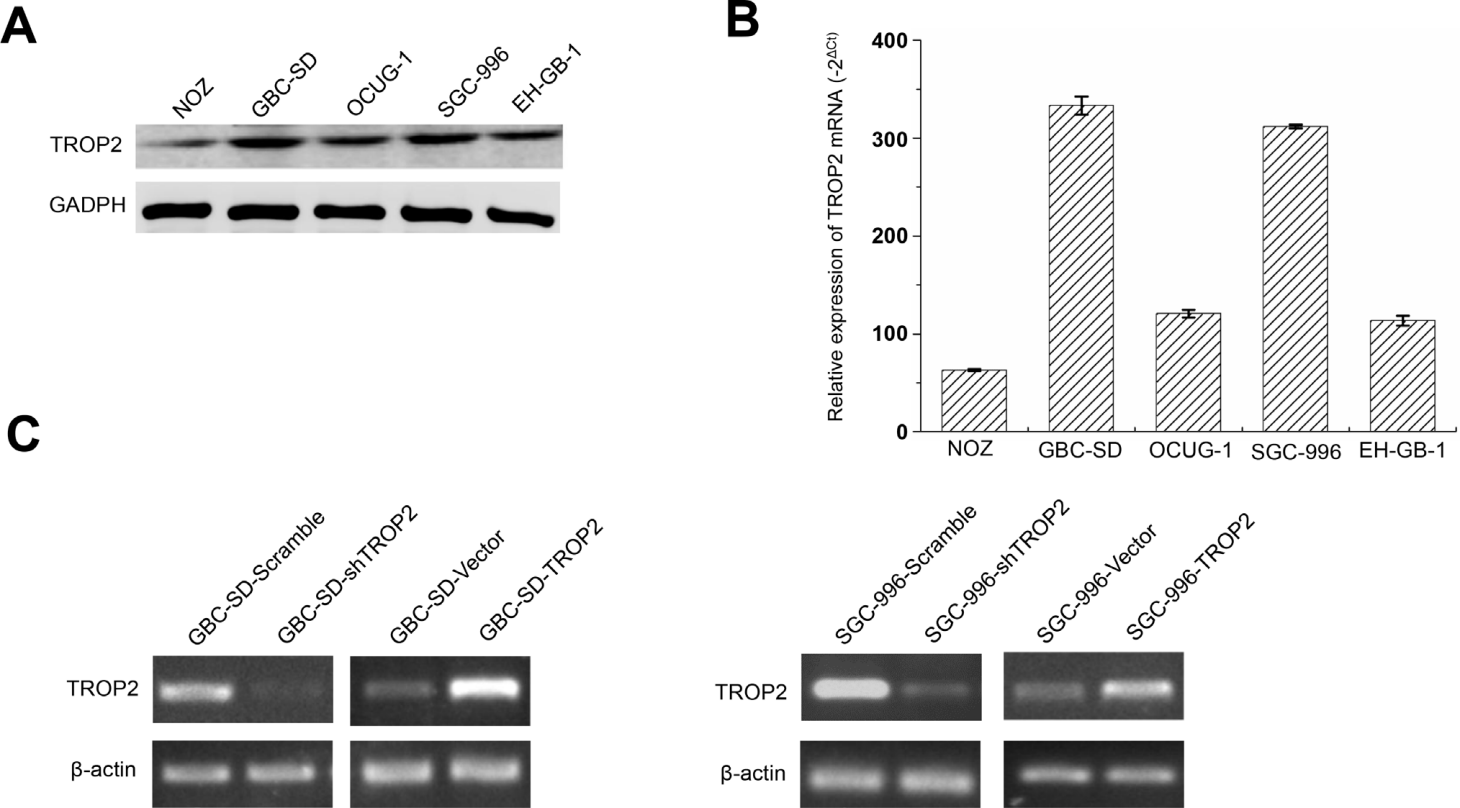
TROP2 expression in GBC cell lines. (**A**) The TROP2 protein expression of human GBC cell lines NOZ, GBC-SD, OCUG-1, SGC-996 and EH-GB-1B. (**B**) The TROP2 mRNA expression of human GBC cell lines NOZ, GBC-SD, OCUG-1, SGC-996 and EH-GB-1B. (**C**) TROP2 mRNA expressions of GBC-SD and SGC-996 cells after RNA interference and plasmid transfection. Cells were transfected with scramble sh-RNA and empty vector as negative controls. Each experiment was repeated three times.

