# Supplementary material for: TROP2 promotes proliferation, migration and metastasis of gallbladder cancer cells by regulating PI3K/AKT pathway and inducing EMT
Source: Oncotarget. 2017 Apr 3;8(29):47052–63. doi: 10.18632/oncotarget.16789 (PMC5564543; doi:10.18632/oncotarget.16789)
Supplement: Supplementary file 1 [file oncotarget-08-47052-s001.pdf]

TROP2 promotes proliferation, migration and metastasis of gallbladder cancer cells by regulating PI3K/AKT pathway and inducing EMT

SUPPLEMENTARY FIGURES AND TABLES

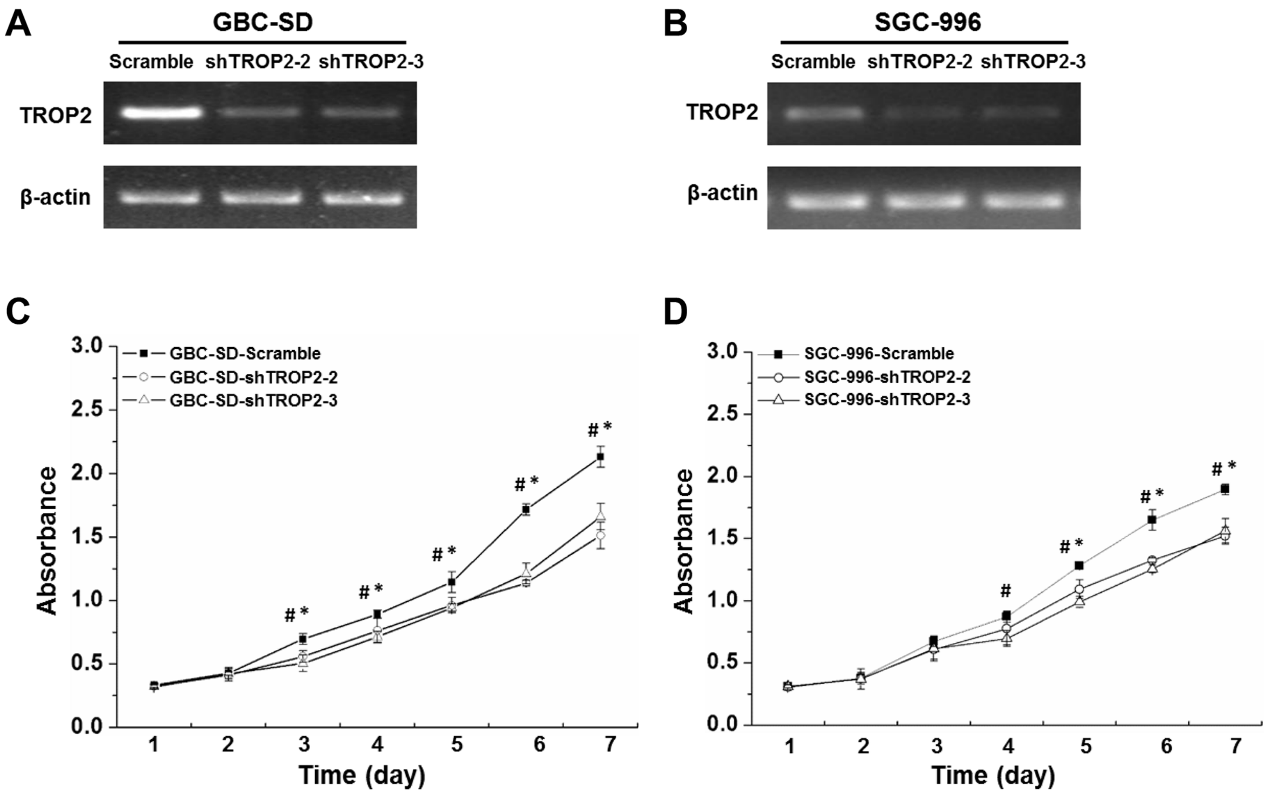

Supplementary Figure 1: TROP2 knockdown using another two sh-RNAs. (**A** and **B**) TROP2 mRNA expression of GBC-SD and SGC-996 cells after RNA interference using shTROP2-2 and shTROP2-3. Cells were transfected with scramble sh-RNA as negative controls. Each experiment was repeated three times. (**C** and **D**) Growth curves of GBC-SD and SGC-996 cells after RNA interference using shTROP2-2 and shTROP2-3. Graphs, mean of three experiments; bars, S.D. *$P < 0.05$, shTROP2-2 group compared with the control group. #$P < 0.05$, shTROP2-3 group compared with the control group.

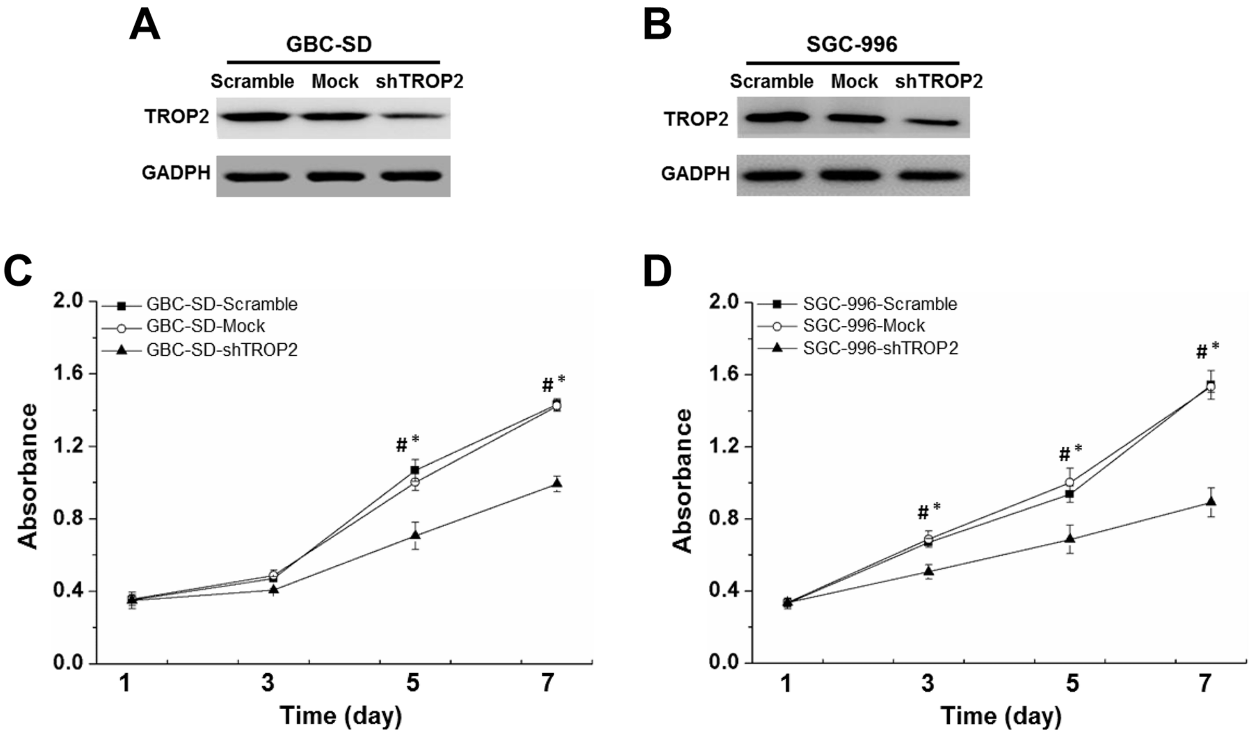

Supplementary Figure 2: Effects of empty vector on proliferation of GBC-SD and SGC-996 cells. (A and **B)** TROP2 protein expression of GBC-SD and SGC-996 cells after empty vector transfection. Cells were transfected with shTROP2 as positive controls. Each experiment was repeated three times. **(C** and **D)** Growth curves of GBC-SD and SGC-996 cells after empty vector transfection. Graphs, mean of three experiments; bars, S.D. $^*P < 0.05$, empty vector group compared with the positive control group. $^\#P < 0.05$, scramble group compared with the control group.

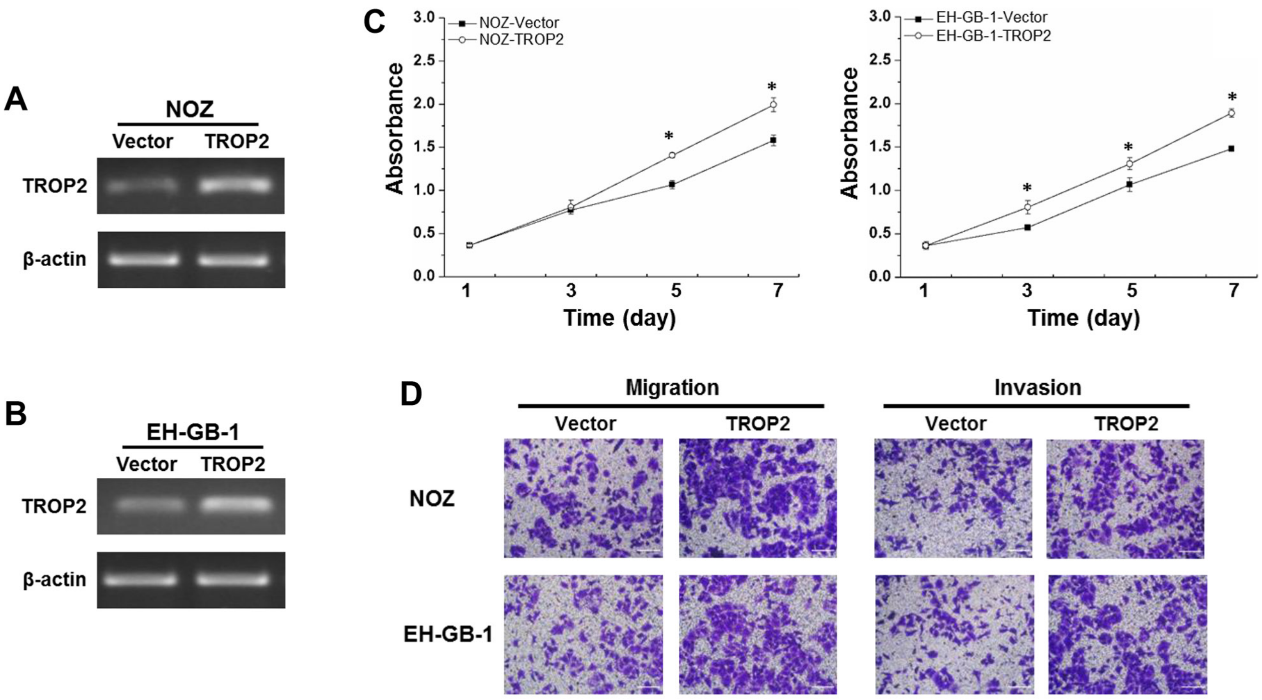

Supplementary Figure 3: Effects of TROP2 on proliferation, migration and invasion of NOZ and EH-GB-1 cells. (A and B) TROP2 mRNA expression of NOZ and EH-GB-1 cells after plasmid transfection. Cells were transfected with empty vector as negative controls. **(C)** Growth curves of NOZ and EH-GB-1 cells after plasmid transfection. Graphs, mean of three experiments; bars, S.D. **P* < 0.05, compared with the control group. **(D)** Microscope images of NOZ and EH-GB-1 cell migration and invasion after TROP2 overexpression. Each experiment was repeated three times.

Supplementary Table 1: Primer pairs

**Primer pairs used for quantitative real-time PCR**	
TROP2	*Forward 5'-CCCCGCGCCTCATCCGCCCGCGTC-3'*
	Reverse 5'-CAAGCTCGGTTCCTTTCTCAACTC-3'
β-actin	*Forward 5'-CTGGAACGGTGAAGGTGACA-3'*
	Reverse 5'-AAGGGACTTCCTGTAACAATGCA-3'
Primer pairs used for RT-PCR	
TROP2	*Forward 5'-TATTACCTGGACGAGATTCCCC3'*
	Reverse 5'-CCCCGACTTTCTCCGGTTG-3'
β-actin	*Forward 5'-GAGACCTTCAACACCCCAGCC-3'*
	Reverse 5'-AGACGCAGGATGGCATGGG-3'
shRNA	
Human gene	Sequence
shTROP2	*Forward 5'-CACCGCCACCAACAAGATGACCGTTTCAAGACGACGGTCATCTTGTTGGTGGTTTTTTG-3'*
	Reverse 5'-AGCTCAAAAAACCACCAACAAGATGACCGTCGTCTTGAAACGGTCATCTTGTTGGTGGC-3'
shTROP2-2	*Forward 5'-CACCAGGGCGAGTCTCTATTCCATTCAAGACGTGGAATAGAGACTCGCCCTTTTTTTG-3'*
	Reverse 5'-AGCTCAAAAAAAGGGCGAGTCTCTATTCCACGTCTTGAATGGAATAGAGACTCGCCCT-3'
shTROP2-3	*Forward 5'-CACCGCACGCTCATCTATTACCTTTCAAGACGAGGTAATAGATGAGCGTGCTTTTTTG-3'*
	Reverse 5'-AGCTCAAAAAAGCACGCTCATCTATTACCTCGTCTTGAAAGGTAATAGATGAGCGTGC-3'
Scramble	*Forward 5'-CACCGCACCTTTCAGCGTGCGGTAATACGCTCATCTATTAGACGAAGATGATTTTTTG-3'*
	Reverse 5'-AGCTCAAAAAAATTAATGAGCGTAATAGAAAAAGCACGCTCCTCGTCTTGAAAGGTGC-3'
